# Agreement between parents and adolescents on emotional and behavioral problems and its associated factors among Chinese school adolescents: a cross-sectional study

**DOI:** 10.1186/1471-244X-14-114

**Published:** 2014-04-15

**Authors:** Jiana Wang, Li Liu, Hui Wu, Xiaoshi Yang, Yang Wang, Lie Wang

**Affiliations:** 1 Department of Social Medicine, School of Public Health, China Medical University, No. 92 Beier Road, Heping District, Shenyang, Liaoning 110001, People’s Republic of China

**Keywords:** School adolescent, Emotional and behavioral problem, Agreement, China, Cross-sectional survey

## Abstract

**Background:**

Most studies about informant agreements on adolescents’ emotional and behavioral problems have been conducted in Western countries, but this subject has not been well researched in China. The aim of this study was to evaluate the pattern of parent–adolescent agreement on adolescents’ problems and its associated factors among school-age adolescents in China.

**Methods:**

This cross-sectional study was conducted in November and December of 2010. A questionnaire including the Child Behavior Checklist (CBCL), the Youth Self-Report (YSR), the Family Environment Scale (FES) and the characteristics of the child (age and gender), parents (parent–adolescent relationship and parental expectations) and family (family structure, negative life events) was distributed to our study population. A total of 2,199 Chinese adolescents (aged 11–18) from 15 public schools in Liaoning Province, who completed the questionnaire, became our final participants. Pearson’s correlation coefficient was used to assess parent–adolescent agreement, and linear regression analysis was used to explore the associated factors of parent–adolescent discrepancies on emotional and behavioral problems.

**Results:**

The parent–adolescent agreement on emotional and behavioral problems was high (mean r = 0.6). The scores of YSR were higher than those of CBCL. Factors that increased informant discrepancies on emotional and behavioral problems were boys, older age, the experience of negative life events, low levels of cohesion and organization, and high levels of conflict in the family.

**Conclusions:**

A high level of parent–adolescent agreement on emotional and behavioral problems was found. Adolescents reported more problems than their parents did. Family environment is an important factor to be considered when interpreting informant discrepancies on the mental health of Chinese adolescents.

## Background

Adolescence is a critical developmental period for emotional and behavioral problems [1]. The World Health Organization states that emotional and behavioral problems in adolescence are an increasing public health concern [2]. Information on adolescents’ problems can be obtained from different informants, including parents and adolescents themselves, and from Achenbach’s series scales, which are widely used around the world when assessing problems in children and adolescents [3,4]. Achenbach’s scales include the Child Behavior Checklist (CBCL), which is completed by parents or teachers [5], and the Youth Self-Report (YSR), which is completed by adolescents themselves [6] to assess emotional and behavioral problems in youth aged 11–18 years. Parents are important informants on their children’s behavioral problems. However, it is unclear the extent to which parents are aware of their children’s diverse behaviors or have the same threshold as their children when rating emotional and behavioral problems [7]. Achenbach suggested that it was necessary to preserve the contributions of different informants and found that different informants reported different levels of problems for adolescents [8]. Therefore, information from both parents and adolescents is needed to obtain a comprehensive assessment of emotional and behavioral problems among adolescents. In China, the parent report is in common use but the adolescent self-report is rarely used. Few studies have collected information from both groups of informants in one survey.

Many investigators have shown specific interest in parent**-**child agreement on adolescents’ emotional and behavioral problems using Achenbach’s scales. Most studies found poor to low CBCL–YSR agreements on Total Problems and Internalizing and Externalizing scales (r range of 0.2–0.4) [3,9,10], and a few studies found moderate to high agreements (r range of 0.5–0.7) [4,11]. A study reviewing cross-informant agreement between parent-reported and adolescent self-reported problems in 25 societies has found that the correlation varied with an r value of 0.27 to 0.65 [12]. The 2013 report by Rescorla et al. of CBCL–YSR cross-informant agreement findings in 25 societies included a large Chinese sample drawn from Wang et al. (2005) [12,13]. As Rescorla et al. reported, the mean cross-informant r averaged across 17 scales was.46 for China [12]. Wang had previously reported correlations ranging from 0.35–0.60 for the same sample (plus 400 dyads excluded from the Rescorla et al. study because of selection criteria) [13]. In addition, it was reported that adolescents reported higher levels of problems than their parents in general population-based studies [12-15], while a reverse pattern occurred in clinical samples [3,9,16,17].

Many studies have examined factors that might influence the level of agreement across informants. Since this study mainly assessed the agreement between parents and self-reports, we wanted to focus on the effects of parental and family factors as well as adolescent characteristics. It has been well established in the literature that both parent and child characteristics were likely to contribute to discrepancies among informants [18-21]. One study found that parents agreed more about the problems of girls than those of boys [7], and conflicting evidence existed regarding the direction of the age effect on informant agreements [10,22,23]. It was reported that the quality of the parent**-**child relationship could also influence the disagreement between parents and their children [19]. One study also found that parental factors correlated with informant disagreements about the level of adolescents’ problems. This was not only because those parents provided an accurate description of increased behavior problems occurring in the home, but also because different parental functions can themselves influence children’s behavior and emotions [21]. One previous study examined negative life events and found that they could have a negative impact on parents’ estimate of children’s problem behaviors [24]. No study took the effect of family environment on the discrepancies into account. It is widely accepted that the family environment has a direct and major influence on adolescents’ behavior [25,26]. Previous studies have concluded that adolescents’ emotional and behavioral problems were consistently related to low levels of family cohesion and high levels of conflict [26,27]. China has the largest adolescent population in the world (283 million adolescents according to the China Yearbook). With the dramatic socioeconomic changes and the increasing social contradictions of the last 30 years, the Chinese family environment has changed dramatically [28]. The Chinese family is confronted with the difficulties of merging traditional ideas (e.g. strict and authoritative) and modern styles (e.g. warm and autonomous) [29]. It has been reported that Chinese adolescents are striving for autonomy from their parents and beginning to view them more as friends [30]. Furthermore, after the implementation of the “One Child Policy” in the 1970s [31], Chinese families have paid more attention to child rearing. All of these changes may have a positive or negative impact on adolescents, especially as Eastern culture requires Chinese adolescents to spend most of their time with their family, whereas adolescents in Western cultures spend more time with their peers [4]. Therefore, Chinese parents may be more familiar with their children and the family environment may greatly influence the understanding parents and adolescents have about adolescents’ problems. Unfortunately, few studies have explored parent–adolescent agreement in China and the associated factors have never involved the family environment. Only one study among Chinese adolescents reported a mean CBCL–YSR correlation of 0.46, which was above the average level among 25 societies when ranked according to the mean cross-informant r value in the summarized findings from the Rescorla study [12]. This study hypothesized that parent–adolescent agreement on adolescents’ emotional and behavioral problems might be moderate, and there would be more parent–adolescent discrepancies when adolescents are in undesirable family environments because these adolescents would be more likely to keep their thoughts from their parents.

The aims of the present study were to: (1) explore the pattern of agreement between parents and adolescents on emotional and behavioral problems with large and representative samples of Chinese adolescents using the CBCL and YSR; and (2) estimate factors associated with informant discrepancies, including the characteristics of the child (age, gender), the parents (parent**-**child relationship and parental expectations) and families (family structure, negative life events and family environment) to provide evidence about the importance of obtaining reports from multiple informants.

## Methods

### Data collection and sample

This study was conducted in Liaoning Province, which is located in the northeast of China, during November/December 2010. Liaoning Province has a population of 48 million and encompasses three metropolitan cities with a population of ≥1,000,000 in each; seven medium-sized cities each having a population of 500,000–1,000,000; and four small cities with a population of 200,000–500,000 each. Three cities (one metropolitan, one medium-sized, and one small) of the 14 cities were randomly selected. From these, three urban areas from each city, and two counties from the large and medium-sized cities, were randomly selected. Counties in small cities with few public schools were not included in this study. Three public schools were randomly selected from each area, including one primary school (grades 5–6) and two senior schools (grades 7–12) according to the age range (11–18 years), and two or three classes were randomly selected from each grade. Ordinarily, there were 50 students in each classroom. We randomly selected 30 students with an equal number of boys and girls aged 11–18 years from each selected class using a pre-prepared list of random numbers. A final total of 2,700 students from 15 public schools were chosen as participants. All the participants and their parents were informed about the content and the aims of this study. After obtaining written consent from the participants to conduct the survey, a questionnaire including the CBCL, YSR and questions about the child, parent and family factors was distributed to 2,700 participants. Of the 2,426 questionnaires returned 227 subjects were excluded because of incorrect information or missing data. The final study subjects consisted of 2,199 Chinese adolescents attending school (921 boys and 1,278 girls). In our population, the average age was 13.0 years old (SD = 2.3, range 11–18 years old). Of the adolescents’ families, 32.3%, 47.3% and 20.4% had low, middle and high socioeconomic status (SES) respectively. In our population, SES was indicated by parents’ highest education level similar to the study of Weine et al. (1995). Education levels were divided into three groups: junior high school level, senior high school level, and college level or higher. The effective response rate was 81.4%. The procedures followed were in accordance with the ethical standards of the Committee on Human Experimentation of China Medical University.

### Measures

#### Child Behavior Checklist (CBCL) and Youth Self-Report (YSR)

The current study used the 1991 versions of the CBCL and YSR because the Chinese 2001 versions of the instruments (on which six of the 1991 items were replaced) were not yet widely available at the time the study was conducted. Therefore, the 1991 versions of the CBCL and YSR syndromes were used in the current study, which differs slightly from the 2001 syndromes in item composition and in names. The CBCL Parent Report Form contains 120 items assessing the emotional and behavioral problems of children 4–18 years old [5]. Each item has three response categories: (0) not true (as far as you know), (1) somewhat or sometimes true, (2) very true or often true. Eight subscales are included: Withdrawn, Somatic Complaints, Anxious/Depressed, Social Problems, Thought Problems, Attention Problems, Delinquent Behavior, and Aggressive Behavior. The Total Problem scale subsumes the eight syndrome scales. The three syndrome scales (Withdrawn, Somatic Complaints and Anxious/Depressed) constitute the broadband Internalizing scale. The syndrome scales Delinquent Behavior and Aggressive Behavior comprise the broadband Externalizing scale. Higher scores denote greater problems. Good reliability and validity of the CBCL were confirmed for the Chinese version [32,33]. The Cronbach’s alpha was 0.98 for the Total Problem scale, 0.96 for the Internalizing scale and 0.95 for the Externalizing scale in this study.

The YSR [6] is used for ages 11–18 to obtain adolescents’ self-reports on their own problems, and the items are worded in the first person. The YSR also includes 120 items similar to those on the CBCL and provides scores for the eight syndrome scales described above and the Total Problem, Internalizing and Externalizing scales. Only those items that CBCL and YSR had in common were used in the analyses. Previous studies supported the good reliability and validity of the Chinese YSR [33,34]. The Cronbach’s alpha was 0.96 in this study. The Cronbach’s alpha was 0.92 for the Internalizing scale and 0.91 for the Externalizing scale.

### Child and parent factors

Child variables included age and gender. In order to make data suitable for the variance analyses, age was used as a categorical variable in this analysis. Age was divided into an “11–14 years old” group and a “15–18 years old” group. Parent factors included the parent–adolescent relationship and parental expectations. The parent–adolescent relationship was measured by asking ‘How did you experience your relationships with your parents?’ with response categories: (1) very close and warm, (2) good, (3) intermediate, (4) somewhat poor, (5) poor. Parental expectations were measured by asking: ‘How high are your parents’ expectations for your learning performance?’ using a four-point scale ranging from high to low expectations.

### Family factors

Family factors included family structure, negative life events and family environment. The classification of family structure included: (1) intact family (living with both biological parents), and (2) non-intact family (living with single parent, step-parent or foster parent) [35]. Negative life events included responding ‘yes’ to any of the following events having occurred in the past year in participants’ families: serious medical problems of the participants, suicidal/violent/criminal behavior of family members, death of extended family members, poor housing, financial problems, death in the nuclear family, theft, accident/disaster or separation from parents. Family environment was assessed using the Family Environment Scale (FES), a widely used and well-validated measure of family characteristics [36]. It has 90 true–false items and includes ten subscales: Cohesion, Expressiveness, Conflict, Independence, Achievement, Active-Recreation, Intellectual–Cultural, Moral–Religious, Organization and Control. Higher scores indicated higher levels of the measure. Seven subscales were used in this study due to the inapplicability of the three excluded subscales of Expressiveness, Independence and Moral–Religious when used in a Chinese context [37]. The Chinese version of this scale has been well documented to have acceptable reliability and validity [38,39]. The Cronbach’s alpha was 0.83 for the total score in this study and 0.42–0.81 for the seven subscales. Only two subscales’ alphas of Achievement and Active-Recreation were lower than 0.70 and Active-Recreation’s alpha was close to 0.7. Either the total score or the individual subscales of FES were taken to have good reliability and validity.

### Statistical analysis

The correlations of the Total Problems, Internalizing and Externalizing scales between CBCL and YSR were analyzed with Pearson’s correlation coefficient. Analyses of variance of repeated measures were used to test the differences between CBCL and YSR and to test the effects and interactions of gender and age on these differences. To measure the parent–adolescent discrepancies, we subtracted CBCL scores from YSR scores for the Total, Internalizing and Externalizing scores, and the absolute values were used as the outcome variables for this study. Higher scores in the absolute values indicated more discrepancies between parents and adolescents. Pearson’s correlation was also used in a univariate analysis to examine the relationship between parent–adolescent discrepancies on emotional and behavioral scores and child, parent and family factors. The categorical variables of gender and family structure were performed by t-tests alone. Linear regression analysis was used to estimate the associated factors of parent–adolescent discrepancies. All variables related to parent–adolescent discrepancies in univariate analysis (P < 0.25) were entered into the model because we wanted to include as many factors as possible in the linear regression analysis. Previous studies also used the changed standard of P value from < 0.05 to < 0.25 in the statistics [40]. They were performed with SPSS for Windows (version 17.0), with a two-tailed probability value of < 0.05 considered statistically significant.

## Results

The mean scores on the Total Problems, Internalizing and Externalizing scales, and correlations of the CBCL and YSR are presented in Table 1. The Cohen’s d values of YSR-CBCL were as follows: YSR-CBCL Total Problems (Cohen’s d = 0.22), YSR-CBCL Internalizing (Cohen’s d = 0.26), and YSR-CBCL Externalizing (Cohen’s d = 0.24).

**Table 1:** Mean (M), standard deviation (SD), and Pearson’s correlation coefficients (r) between CBCL and YSR in total problems, internalizing and externalizing scales (n = 2199)

	**CBCL**	**YSR**	**r**
	**M (SD)**	**M (SD)**	
Total	24.61 (30.0)	30.03 (23.7)	0.593***
Internalizing	9.68 (12.1)	12.35 (10.8)	0.611***
Externalizing	7.02 (9.6)	8.91 (8.6)	0.614***

*P < 0.05, **P < 0.01, ***P < 0.001.

Table 2 shows the percentages (effect sizes) of variance. The mean problem scores for YSR were significantly higher than for CBCL on the Total Problems, Internalizing and Externalizing scales. The effect sizes of interactions between the CBCL-YSR discrepancy scores and gender or age were all very small (<1).

**Table 2:** Percentage of variance of significant effects in analyses of variance of mean problem scores

**Variable**	**Informant**	Informant*gender	Informant*age	Informant*age*gender
Total	9.6***	—	—	—
Internalizing	9.3***	< 1*	—	—
Externalizing	5.1***	< 1	< 1**	< 1**

Effect size according to Cohen’s (1977) standards [41]: for 1–5.9% of variance are small; 5.9-13.8% are medium, and above 13.8% are large. Less than 1% are indicated < 1.

*P < 0.05, **P < 0.01, ***P < 0.001.

— Non-significant effect.

Most adolescents (89.9%) perceived that their parents had high expectations for them, and 89.5% adolescents had good relationships with their parents. The majority (90.6%) of the adolescents were living in intact families and slightly over half (51.3%) were living in families who had experienced one or more negative life events. The average scores of subscales for FES were as follows: Cohesion (M = 7.56, SD = 1.93), Conflict (M = 2.25, SD = 1.94), Achievement (M = 5.78, SD = 1.74), Intellectual-Cultural (M = 5.09, SD = 2.04), Active-Recreation (M = 5.24, SD = 2.23), Organization (M = 6.57, SD = 2.09), and Control (M = 3.91, SD = 1.92).

The results of univariate analyses between the CBCL-YSR discrepancy scores and all variables are shown in Table 3. All indicators were significantly related to the YSR-CBCL discrepancy scores (P < 0.05) except for family structure, and control for all the scales. Parental expectations were not found to be significant for the Internalizing scale. Gender had only a small but significant effect on Total Problems (boys > girls) in CBCL-YSR discrepancy scores (P < 0.05).

**Table 3:** Pearson’s correlation coefficients between CBCL-YSR discrepancies and related factors

	**Discrepancy CBCL-YSR**		
	**Total**	**Internalizing**	**Externalizing**
Age (year)	0.138***	0.134***	0.228***
Parent-adolescent relationship	0.126***	0.141***	0.123***
Parent expectation	0.050***	0.041	0.084***
Negative life event	0.150***	0.157***	0.134***
Cohesion	−0.238***	−0.220***	−0.245***
Conflict	0.251***	0.239***	0.255***
Achievement	−0.095***	−0.082***	−0.102***
Intellectual-Cultural	−0.104***	−0.105***	−0.121***
Active-Recreational	−0.107***	−0.119***	−0.102***
Organization	−0.230***	−0.212***	−0.253***
Control	0.016	0.008	0.008

*P < 0.05, **P < 0.01, ***P < 0.001.

The results of the linear regression analysis for discrepancies on the Total Problems, Internalizing and Externalizing scales are shown in Table 4. Only the significant variables are presented (P < 0.05). The percentages of variance accounted for by all the associated factors were 10.8% for the Total Problems, 8.8% for Internalizing and 12.3% for Externalizing scales.

**Table 4:** Results of linear regression between CBCL-YSR discrepancies and associated factors

**Variable**	**B**		
	**Total**	**Internalizing**	**Externalizing**
Gender (girl versus boy)	−0.05*		
Age	0.09***	0.069**	0.164***
Negative life event	0.05*	0.114***	0.078***
Cohesion	−0.09**	−0.073*	−0.061*
Conflict	0.15***	0.133***	0.129***
Organization	−0.10***	−0.075**	−0.120***

*P < 0.05, **P < 0.01, ***P < 0.001.

A positive coefficient indicates that the discrepancy increases.

A negative coefficient indicates that the discrepancy decreases.

## Discussion

This study estimated both the pattern of agreement between parents and adolescents on emotional and behavioral problems, and its associated factors among Chinese adolescents aged 11–18 years. A fairly large sample constituted by students from grades 5–12 of schools in each city size, including urban and rural areas in Liaoning province, was used. The effective response rate was 81.4%, which was higher than the norm of 70% for questionnaire surveys [42]. Thus, our study population was representative of the population, which increases the generalizability of our study conclusions.

Our results found a high parent–adolescent agreement on emotional and behavioral problems (mean r = 0.6), which was a little higher than we had expected. There may be numerous reasons why parent–adolescent agreement was higher in our Chinese sample than in many previous samples. First, it may be because of the special pattern of family life that exists in China. Chinese families, deeply influenced by traditional culture, are regarded as the most important living environment, and adolescents depend on their parents. The relationship between parents and adolescents is closer than that in Western countries [4]. In our subjects, most adolescents (89.5%) had good parent–adolescent relationships (‘very close and warm’ and ‘good’) and most families in China have only one child (85.3% of our subjects were only children). Because of this, parents may pay more attention to their child and better understand their behavior and problems. Second, parent–adolescent discrepancies may be smaller in societies where cultural values promote familism and collectivism, such as those with Confucian or Catholic traditions, than in societies that promote individualism and autonomy [12]. However, one research study reported a lower mean correlation of 0.46 between the CBCL and YSR in a sample of 1,022 participants in China [13] even though the correlation was higher than that in most other countries. This result might reflect the dramatic change of the Chinese family environment in recent decades to one where the way of rearing children has become more democratic than in the past and where the generation gap is reducing [33]. The regional difference, despite lack of supporting evidence, might also explain more or less of the different agreement levels found. As Beijing is the capital, it is one of the most developed areas of China. People might have more opportunities to be exposed to and to accept new cultural norms. Adolescents here might be more independent and less willing to be controlled by their family. Liaoning, in contrast, is located inland in the northeast and has average economic and population levels. People may have deeper traditional views and stronger orientation to familism even though westernization has also influenced their lives. Therefore, parents may be closer and more familiar with their children, and their agreement with adolescents’ problems is higher than that in Beijing as a result. Further research should explore regional differences. Parent–adolescent agreement was relatively higher in our study than in other countries and in earlier Chinese research.

Our study also revealed that adolescents reported more problems than their parents on all the problems studied. Most previous studies reported consistent results in the general population [14,15], but contrary results occurred in the clinical sample [3,9,16,17]. This might be due to various reasons. In the general population, some problems of adolescents would be hidden from their parents’ view [24]. In addition, parents might conservatively assess the problems of their children because they do not expect them to be unwell, whereas in a clinical sample, parents hope for healing and therefore observe their child’s emotions and behaviors closely [7]. Rescorla et al. also reported that YSR scores were higher than CBCL scores among the general population in all 25 societies [12]. The difference was the informant effect sizes (ES) of our study, which were smaller than those Rescorla et al. reported. The Cohen d values of YSR-CBCL were also quite small, according to the study of Cohen (1988) [43]. The reason for the total large ES from 25 societies in different culture was uncertain. As mentioned above, parent–adolescent discrepancies may be smaller in societies such as China, which are characterized by familism and collectivism cultural values. It is worth mentioning that, as the Rescorla (2013) study clearly indicates, the mean YSR > CBCL discrepancy fails to capture the fact that in many studies dyad CBCL scores are higher than YSR scores. This is a highly consistent finding across societies. Therefore, both parents and adolescents are needed to obtain a comprehensive picture of adolescents’ mental health and it was important to obtain reports from multiple informants.

This study also contributed to understanding the associated factors of parent–adolescent discrepancies. Previous research on discrepancies had mainly focused on adolescents’ age and gender [18-20]. Although the ES was small for both age and gender (<1%), our findings also found these effects, which indicated that boys had larger discrepancies than girls possibly because boys are more guarded about communicating their feelings and problems at home [7]. However, as with age, the findings were inconsistent. Some studies have reported that discrepancies decrease with increased age [10,22]. Our study found that older adolescents had larger discrepancies than younger ones, which is consistent with other studies [23]. Perhaps as adolescents grow older, they increasingly keep their feelings and behaviors to themselves [44]. Future research is needed to explore this association. Negative life events were also found to have an impact on this discrepancy. Parents living in families encountered more life events and agreed less with their children than those in families who underwent fewer negative events. This finding was in accordance with a previous study, which reported that negative life events were associated with parents’ perceptions of their children’s problem behavior [24].

An important finding of this study was that family environment had a strong effect on discrepancies in the Total Problems, Internalizing and Externalizing scales, which supported the hypothesis that there would be more parent-adolescent discrepancies when adolescents are in undesirable family environment. This was the first attempt to evaluate the influence of family environment on parent–adolescent discrepancies regarding adolescents’ emotional and behavioral problems. This finding showed that parents and adolescents living in undesirable family atmosphere such as those with decreased cohesion and organization, and increased conflict tended to report more disagreement. There may be several reasons for this. Family environment may be the key factor in influencing mental health for adolescents [25,26]. A recent study reported that family environment was related to children’s behavioral self-control and emotional regulation [26]. This indicated that adolescents in better family environments might more regularly express their emotional expressions and their behavior might be more easily observed. Thus, these parents might be more familiar with their children’s behaviors and emotion, and this may have contributed to less discrepancy. Moreover, family environment may have a direct impact on parent–adolescent conflicts. Some studies have found that parents and children in harmful family environments experience more divergence and conflicts. Conversely, less divergence has been found in warm and supportive families [45]. Moreover, our population had relatively higher cohesion and organization, and lower conflict than found in Western populations [36]. The reason for these differences might be that most adolescents (90.6%) in our study population lived with both parents, and this might have increased their chances of contact with their parents and established a good parent–adolescent relationship. Moreover, China has a generally collectivist culture, which might generate higher family organization in contrast with the individualism of Western societies [46]. All of these characteristics might also be the reason that our study population showed relatively higher parent–adolescent agreement. These findings suggest that high family cohesion and organization, together with low conflict, may be important targets for parents who need help in understanding more about their children. They may, therefore, provide some indication to clinicians and health care providers when considering the family environment in screening and helping the problem child.

Several limitations of the present study must be mentioned. The first limitation is the cross-sectional design. It is impossible to draw causal conclusions from information obtained in this study, and longitudinal studies should be carried out in the future to confirm these findings. Second, the informants were requested to complete the questionnaires without consulting each other. However, it may have been hard to avoid communication with each other, which might have affected informant agreement. Despite these limitations, we believe that these findings provide important information about parent–adolescent agreement on Chinese adolescents’ mental health.

## Conclusions

Our findings indicated important differences in patterns of agreement between Chinese adolescents and their parents with regard to the adolescent’s behavioral and emotional problems relative to findings reported from Western cultures. Our study revealed that parent–adolescent agreement was greater than that in most previous studies, and adolescents reported more problems than parents. The characteristics of the adolescent, parents and families were associated with the parent–adolescent discrepancies on adolescents’ emotional and behavioral problems. Family environment was a crucial factor which was not taken into account previously. These findings suggest that Chinese parents have a relatively good agreement with their children but tend to underreport their children’s problems. Clinicians and health care providers should pay attention to the family environment when aiming to help adolescents with emotional or behavioral problems.

## Competing interests

None identified. We confirm that the content of this paper is currently not submitted or published in full or in part elsewhere.

## Authors’ contributions

J-NW, LL, HW, XY, YW and LW carried out the investigation among adolescents in school. J-NW thematically analyzed the data and collaboratively wrote the manuscript. J-NW and LL edited the manuscript for submission. LW conceived the study, and supervised its design and data analysis. All authors read and approved the final manuscript.

## Pre-publication history

The pre-publication history for this paper can be accessed here:

http://www.biomedcentral.com/1471-244X/14/114/prepub
